# External Validation of the PRECISE‐DAPT Cancer Score in Patients With Acute Myocardial Infarction

**DOI:** 10.1002/ccd.70040

**Published:** 2025-07-22

**Authors:** Mohamed Dafaalla, Francesco Costa, Evangelos Kontopantelis, Rodrigo Bagur, Mario Iannaccone, Paolo Bironzo, Sergio Raposeiras Roubí, Ovidio De Filippo, Fabrizio D'Ascenzo, Mamas A. Mamas

**Affiliations:** ^1^ Keele Cardiovascular Research Group, Centre for Prognosis Research Keele University Stoke‐on‐Trent UK; ^2^ Cardiology Department University Hospital Virgen de la Victoria, Instituto de Investigación Biomédica de Málaga (IBIMA) Málaga Spain; ^3^ Centro de Investigación Biomédica en Red en Enfermedades Cardiovasculares (CIBERCV) Instituto de Salud Carlos III Madrid Spain; ^4^ National Institute for Health Research School for Primary Care Research, Division of Population Health, Health Services Research and Primary Care, School of Health Sciences, Faculty of Biology, Medicine and Health, Manchester Academic Health Science Centre University of Manchester Manchester UK; ^5^ London Health Sciences Centre Western University London Ontario Canada; ^6^ Division of Cardiology San Giovanni Bosco Hospital Torino Italy; ^7^ Department of Oncology, San Luigi Hospital University of Turin Orbassano Italy; ^8^ Department of Cardiology Hospital Álvaro Cunqueiro Vigo Spain; ^9^ Division of Cardiology, Cardiovascular and Thoracic Department “Città della Salute e della Scienza” Hospital Turin Italy; ^10^ Department of Medical Sciences University of Turin Turin Italy; ^11^ National Institute for Health and Care Research (NIHR) Birmingham Biomedical Research Centre Birmingham UK

**Keywords:** AMI, PRECISE‐DAPT cancer score, validity

## Abstract

**Aims:**

We aimed to externally validate the PRECISE‐DAPT cancer score which showed better accuracy in predicting bleeding events in patients with cancer than the original PRECISE‐DAPT score.

**Methods:**

We used data from the BleeMACS (Bleeding complications in a Multicenter registry of patients discharged after an Acute Coronary Syndrome) project. We compared the performance and clinical usefulness of the original score and the cancer score by calculating the C‐statistic, the net reclassification index (NRI), and decision curve analysis.

**Results:**

A total of 13,932 patients were included, of which 864 patients had a diagnosis of cancer at the time of presentation with an AMI. According to the original PRECISE DAPT score, 63.3% of patients with cancer were classified as HBR, whereas 94.9% of patients with cancer were classified as HBR according to the cancer score. Cox‐regression models showed that patients classified as HBR by the updated cancer score have higher odds of bleeding (HR 2.6, 95% CI 2.1−3.1) events than patients classified as HBR by the original score (HR 2.2, 95% CI 1.8−2.7). The cancer score showed higher discrimination ability (C‐statistic 0.66) than the original score (C‐statistic 0.64). The overall NRI of the cancer score was 2.7%. The decision curves analysis showed that the cancer score use is roughly identical to the original score in patients without cancer but superior to the original score in patients with cancer.

**Conclusion:**

The PRECISE‐DAPT cancer score is a valid and useful tool for the prediction of bleeding risk in patients with cancer and presenting with AMI.

## 1 Introduction

Cardiovascular diseases (CVDs) and cancer are the main causes of hospitalization and mortality in developed countries [1]. The number of cancer survivors has increased secondary to remarkable improvements in cancer screening and treatment [2]. As a result, patients with cancer are now expected to survive longer and many will develop CVDs due to shared risk factors such as underlying inflammatory processes and cardiotoxicity of cancer treatment. In fact, concurrent evidence shows that patients with cancer are now more likely to die from CVDs rather than the primary cancer itself [3, 4]. Currently, acute myocardial infarction (AMI) is the most common cause of CV mortality in patients with cancer and around 10% of AMI patients who receive percutaneous coronary intervention (PCI) have a current or prior history of cancer [5, 6, 7].

Dual antiplatelet therapy (DAPT) has a key role in the prevention of ischemic events in patients presenting with AMI or undergoing PCI [8, 9, 10]. While DAPT therapy reduces ischemic events risk, it can increase the risk of bleeding complications, particularly in patients at high bleeding risk (HBR). Current guidelines have recommended classification of patients with active malignancy as HBR patients [11, 12], although previous work has shown that many cancer types are not associated with increased risk of bleeding events [13, 14]. Risk prediction tools can be useful in objectively identifying patients with cancer who would benefit from DAPT without increasing the risk of major bleeding. The PRECISE‐DAPT score has been adopted by clinical guidelines to guide the duration of antiplatelet therapy in patients following PCI, where patients are deemed to be at HBR [15]. One limitation of the PRECISE‐DAPT score is that cancer, known to increase the risk of bleeding events, is not considered in its calculation. We recently developed an updated score, the PRECISE‐DAPT cancer score, where cancer was added as a feature to the score [16]. The internal validation of the PRECISE‐DAPT cancer score showed that it has better accuracy in predicting bleeding events in patients with cancer than the original PRECISE‐DAPT score [16].

External validation of the PRECISE‐DAPT cancer score is important to confirm the score's ability to predict bleeding events allowing clinicians to use a simple valid tool to make objective decisions about bleeding risk in patients with cancer. Therefore, we externally validated the PRECISE‐DAPT cancer score in a data set from the BleeMACS (Bleeding complications in a Multicenter registry of patients discharged after an Acute Coronary Syndrome) project.

## 2 Methods

### 2.1 Study Population

We included 13,932 patients from the BleeMACS project. The design and patient population of the BleeMACS registry was comprehensively described previously [17, 18]. Briefly, the BleeMACS project was a multicentre cohort study involving consecutive adult patients presenting with AMI. Participants were recruited from 15 hospitals, from Europe (Germany, Poland, Netherlands, Spain, Italy, and Greece), North and South America (Canada and Brazil), and Asia (China and Japan). The data was collected between November 2003 and June 2014 [17]. The ethical approval was obtained by each center's ethical committee [17]. Patients were systematically followed for 1 year after discharge to assess mortality and bleeding complications which was ascertained by trained research coordinators at each participating site. Data about bleeding were obtained from hospital records, by contacting the patients or their relatives by phone, or by contacting the primary care physicians. For patients treated for adverse events at other medical institutions [17].

### 2.2 Outcomes

The aim of this study is to externally validate the PRECISE‐DAPT cancer score. A bleeding event was defined as any bleeding event requiring hospitalization and/or red cell transfusion concentrates within 1 year.

### 2.3 The Original PRECISE‐DAPT Cancer Score

The simplified version of the original PRECISE‐DAPT score is a 4‐item score which was developed from a pooled data set of eight randomized studies including 14,963 patients. The variables included were age, prior hemorrhage, creatinine clearance, and hemoglobin levels. The difference with the full 5‐item version of the score was the removal of the white cell count which was the weakest predictor [15]. The 4‐item version of the score has been extensively validated, confirming similar prediction performance compared to the original 5‐item score, and is used to inform DAPT duration treatment decisions [15, 19, 20]. A more detailed description of the generation of the PRECIS‐DAPT and mini‐PRECISE‐DAPT scores is available in previous publications [15, 19].

The PRECISE‐DAPT cancer score is a 5‐item score generated by adding cancer as a binary variable to the simplified PRECISE‐DAPT score [16]. It was developed using a Cox regression model based on the following predictors: age, prior hemorrhage, creatinine clearance, hemoglobin levels, and cancer (binary variable). The regression coefficients were used to generate an overall score which was then scaled from 0 to 100, with the higher values indicative of a higher risk of bleeding. Patients were then classified into four bleeding risk categories (very low, low, moderate, and HBR, categories based on the following cut‐offs: less than or equal to 5, 6−14, 15−24, and ≥ 25, respectively) [15, 19]. The PRECISE‐DAPT cancer score was developed and internally validated on a prospectively collected data set from the UK Myocardial Infarction National Audit Project (MINAP). MINAP registry captures data on the presentation profile and clinical care of patients hospitalized with the diagnosis of AMI in England, Wales, and Northern Ireland [21, 22, 23, 24, 25]. The internal validation of the PRECISE‐DAPT cancer score showed higher discrimination ability than the original PREICISE‐DAPT score [16].

### 2.4 Risk Score Performance Assessment

We tested the performance of the original PRECISED‐DAPT score and the PRECISE‐DAPT cancer score assessing its discrimination and calibration capacity by calculating the C statistic and the net reclassification index (NRI). A calibration curve was generated by comparing observed against predicted probabilities. The clinical usefulness and net benefit were estimated with decision curve analysis. Decision curve analysis calculates a clinical “net benefit” for one or more prediction models or diagnostic tests in comparison to default strategies of treating all or no patients. Net benefit is calculated using a range of threshold probabilities, defined as the minimum probability of disease at which further intervention would be warranted, as net benefit = sensitivity × prevalence–(1–specificity) × (1–prevalence) × w where w is the odds at the threshold probability [26]. The statistical analysis was performed using Stata v16 software.

## 3 Results

### 3.1 Patients' Characteristics

A total of 13,932 patients from the BleeMACS registry were included, of which 864 patients had a diagnosis of cancer at the time of presentation with an AMI. Patients with cancer were older (median age 73.0 [IQR 65.0, 79.0] vs. 63.0 [IQR 54.0, 73.0]) and more likely to present with a non‐ST‐elevation myocardial infarction (NSTEMI) (49.5% vs. 43.5%). Patients with cancer were also less likely to receive PCI (55.1% vs. 60.5%) and DAPT (92.8% vs. 95.7%). At 1 year, patients with cancer had a higher rate of death (11.8% vs. 3.3%), reinfarction (8.3% vs. 3.7%), and bleeding (6.7% vs. 3.0%). Table 1 shows the patients' characteristics.

**Table 1:** Baseline characteristics.

	No cancer	Cancer	*p* value
*N*	13,068	864	
Age, median (IQR)	63.0 (54.0, 73.0)	73.0 (65.0, 79.0)	< 0.001
Male	10,090 (77.2%)	614 (71.1%)	< 0.001
Female	2978 (22.8%)	250 (28.9%)	
NSTEMI/unstable angina	5666 (43.4%)	428 (49.5%)	< 0.001
STEMI	7402 (56.6%)	436 (50.5%)	
Diabetes	3134 (24.0%)	251 (29.1%)	< 0.001
Hypertension	7578 (58.0%)	572 (66.2%)	< 0.001
Hyperlipidaemia	6707 (51.3%)	417 (48.3%)	0.081
Peripheral vascular disease	768 (5.9%)	99 (11.5%)	< 0.001
Prior AMI	1578 (12.1%)	136 (15.7%)	0.001
Prior PCI	1581 (12.1%)	124 (14.4%)	0.050
Prior CABG	436 (3.3%)	45 (5.2%)	0.004
History of stroke	764 (5.8%)	76 (8.8%)	< 0.001
History of heart failure	319 (2.8%)	42 (5.7%)	< 0.001
Chronic kidney disease	145 (3.1%)	19 (6.7%)	< 0.001
Femoral access	6774 (57.3%)	447 (59.8%)	0.19
Multivessel disease	4863 (48.3%)	325 (48.5%)	0.91
DES	5518 (42.2%)	311 (36.0%)	< 0.001
PCI without stent	472 (3.6%)	52 (6.0%)	< 0.001
Thrombolysis	224 (1.7%)	9 (1.0%)	0.14
Complete revascularization	6528 (60.5%)	384 (55.1%)	0.005
Hemoglobin, median (IQR)	13.7 (12.4, 14.8)	12.5 (11.1, 13.9)	< 0.001
Creatinine clearance, median (IQR)	83.0 (68.6, 100.7)	76.6 (57.3, 94.2)	< 0.001
Medications			
Aspirin	12,915 (98.8%)	848 (98.1%)	0.077
Clopidogrel	11,409 (87.3%)	779 (90.2%)	0.014
Ticagrelor	580 (4.4%)	20 (2.3%)	0.003
Prasugrel	640 (4.9%)	17 (2.0%)	< 0.001
Oral anticoagulation	685 (5.2%)	66 (7.6%)	0.003
Beta blockers	10,671 (81.7%)	645 (74.7%)	< 0.001
ACEI_ARB	9851 (75.4%)	611 (70.8%)	0.002
Statins	12,205 (93.4%)	783 (90.6%)	0.002
PPI	5220 (55.1%)	424 (67.1%)	< 0.001
DAPT	12,509 (95.7%)	802 (92.8%)	< 0.001
Triple therapy	527 (4.0%)	53 (6.1%)	0.003
Outcomes			
Death at 1 year	430 (3.3%)	102 (11.8%)	< 0.001
Reinfarction at 1 year	414 (3.7%)	65 (8.3%)	< 0.001
Bleeding at 1 year	396 (3.0%)	58 (6.7%)	< 0.001

### 3.2 Bleeding Risk

**Figure 1:**
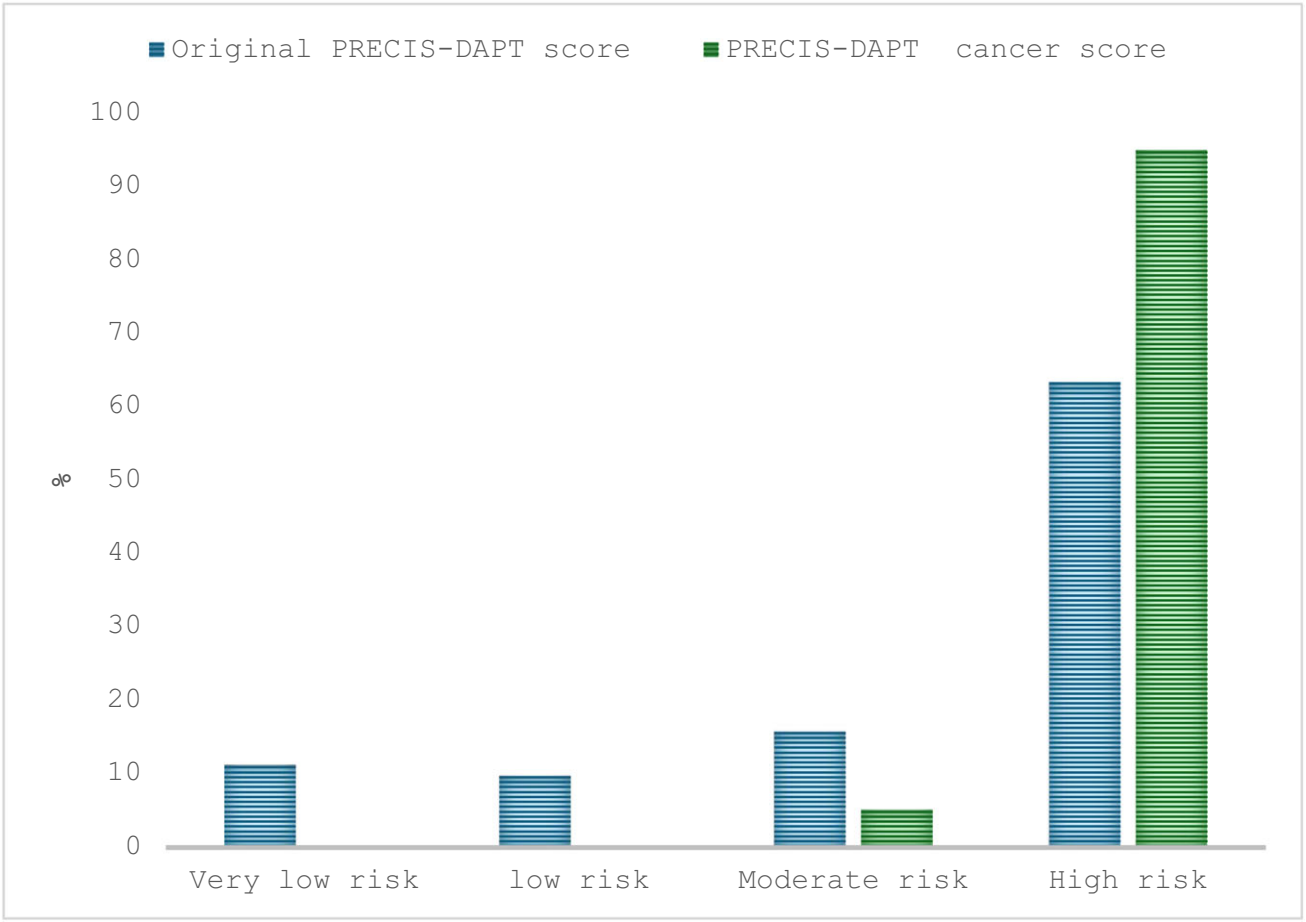
Bleeding risk in patients with cancer according to the original and the PRECISE‐DAPT cancer score. [Color figure can be viewed at wileyonlinelibrary.com]

**Figure 2:**
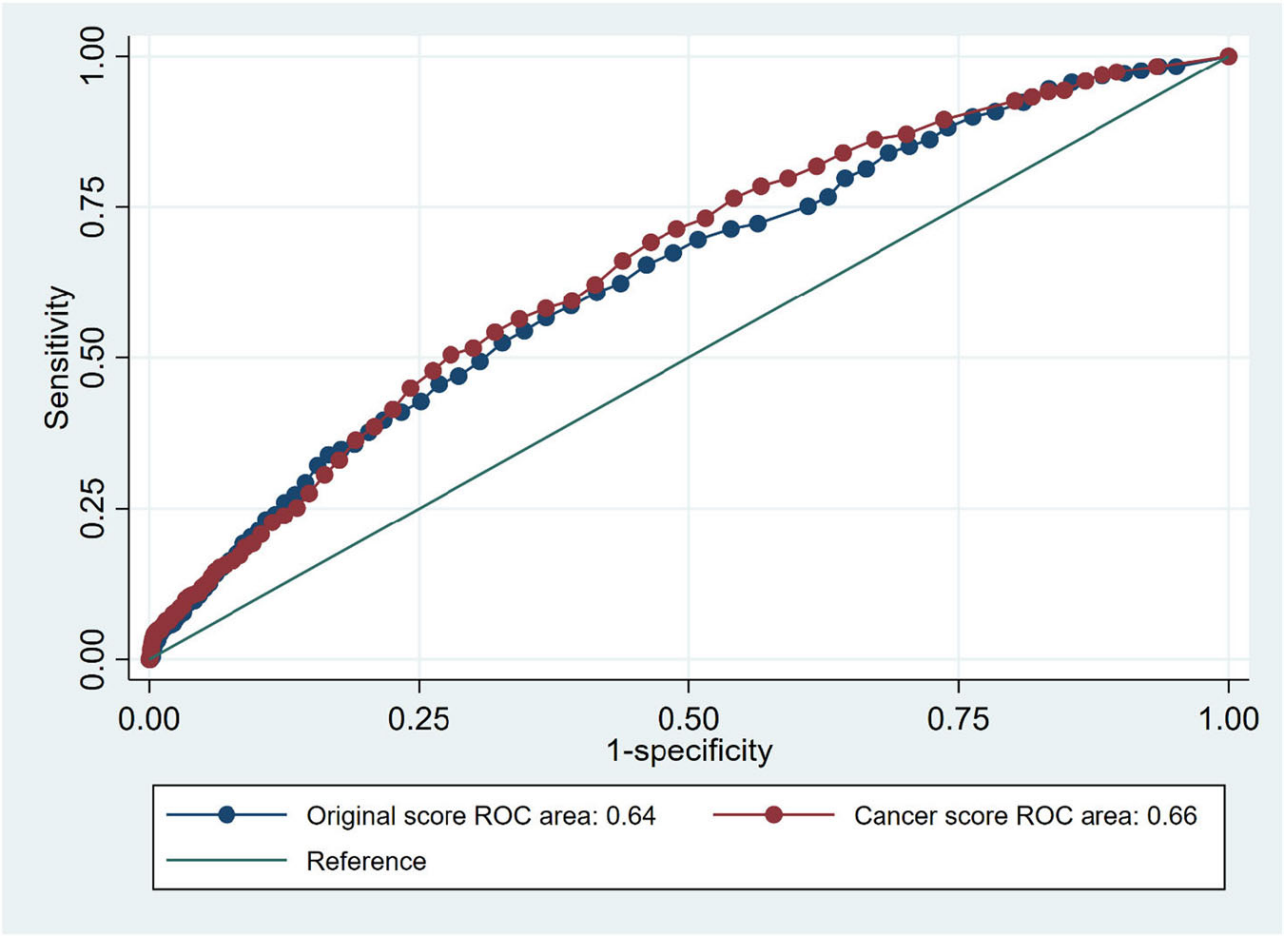
ROC curves of the original PRECISE‐DAPT score and the PRECISE‐DAPT cancer score. [Color figure can be viewed at wileyonlinelibrary.com]

Figure 1 shows the bleeding risk categories in patients with cancer according to the original PRECISE‐DAPT score and the PRECISE‐DAPT cancer score. According to the original score, 63.3% of patients with cancer were classified as HBR, and 20.9% were considered to have a low or very low bleeding risk. According to the PRECISE‐DAPT cancer score, 94.9% of patients with cancer were classified as HBR, 5.1% were classified as moderate bleeding risk, and no cancer patient was classified as low or very low bleeding risk. Supporting Information S1: Figure 1 shows the corresponding HR of bleeding of the PRECISE DAPT original score and the PRECISE DAPT cancer score. Supporting Information S1: Figure 2 shows the cumulative incidence of bleeding events according to the PRECISE‐DAPT cancer score categories.

### 3.3 Performance of the Original PRECISE‐DAPT Score and the PRECISE‐DAPT Cancer Score

**Figure 3:**
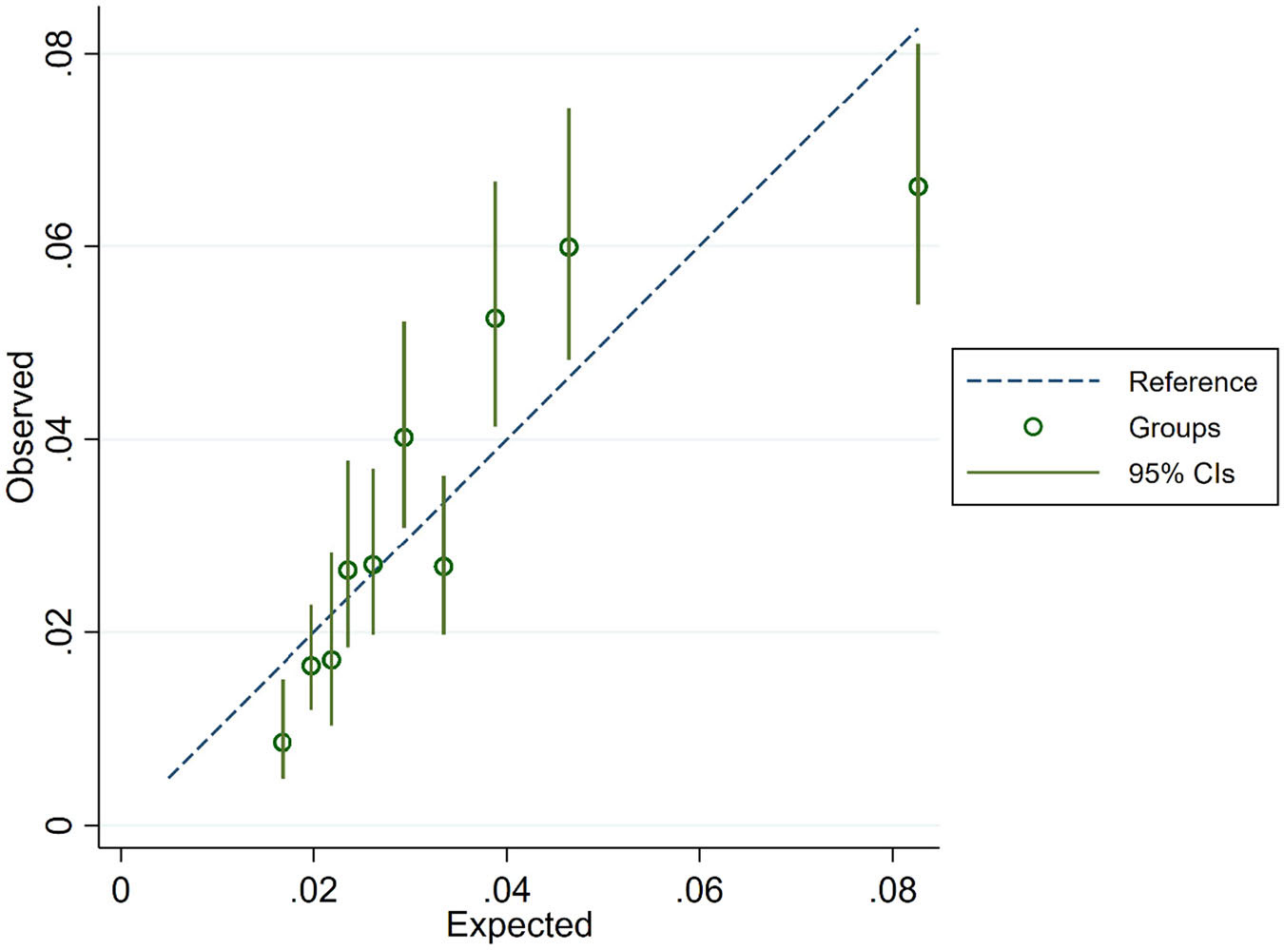
Calibration curve for the PRECIS‐DAPT cancer score. [Color figure can be viewed at wileyonlinelibrary.com]

We used the BleeMACS registry data set to compare the ability of the original score and PRECISE‐DAPT cancer score to predict bleeding events within 1 year post‐discharge. Cox regression models showed that patients classified as HBR by the cancer score have higher odds of bleeding (HR 2.6, 95% CI 2.1−3.1) events than patients classified as HBR by the original score (HR 2.2, 95% CI 1.8−2.7). The cancer score showed higher discrimination ability (C‐statistics 0.66) than the original score (C‐statistics 0.64) improving score specificity from 58.6% to 67.2% (Table 2). Figure 2 shows the ROC curves of the original PRECISE‐DAPT score and the PRECISE‐DAPT cancer score. The overall NRI of the cancer score was 2.8% (Table 3). Figure 3 shows the calibration curve of the modified score; the calibration slope is 1, and the calibration in‐the‐large (CITL) is zero.

**Table 2:** Performance of the original and the PRECISE‐DAPT cancer score.

Original PRECISE‐DAPT score	
C‐statistics	0.64
Sensitivity	60.8%
Specificity	58.6%
Positive predictive	4.7%
Negative predictive	97.8%
**PRECISE‐DAPT cancer score**	
C‐statistics	0.66
Sensitivity	54.2%
Specificity	67.2%
Positive predictive	5.4%
Negative predictive	97.8%

**Table 3:** Net reclassification index of the cancer score.

Risk category (HBR vs. not HBR)	Bleeding at 1 year		
	No	Yes	Total
Downgraded	1936	55	1991
No change	10,872	374	11,246
Upgraded	670	25	695
Total	13,478	454	
NRI			2.80%

### 3.4 Decision Curve Analysis

**Figure 4:**
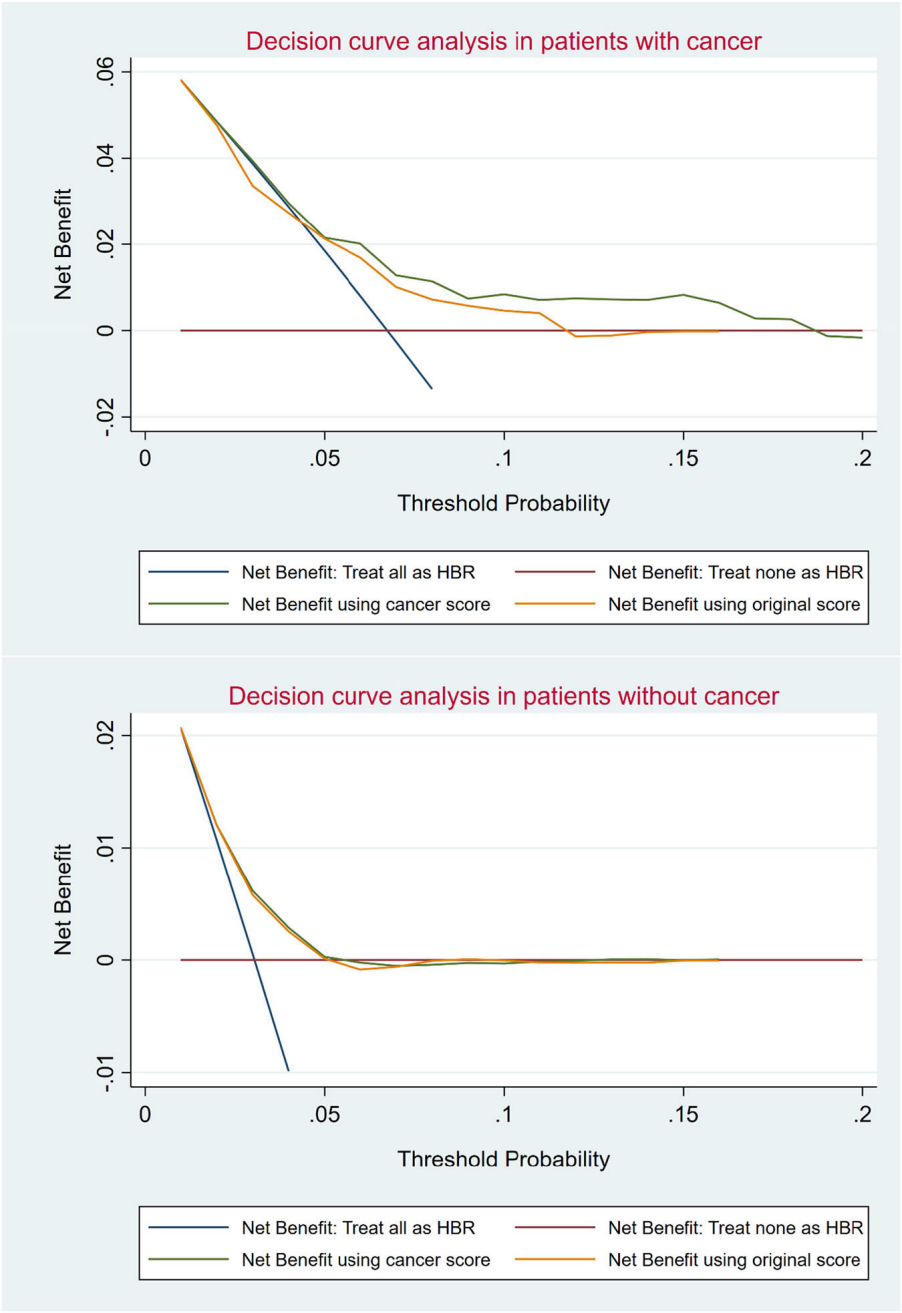
Decision curves for the original PRECISE‐DAPT score and the PRECISE‐DAPT cancer score. [Color figure can be viewed at wileyonlinelibrary.com]

Figure 4 compares the decision curves from classifying individuals using the PRECISE‐DAPT cancer score, assuming all patients as if they will bleed (all positive or all are at high risk of bleeding), and assuming all patients as if none will bleed (all negative or all are at low risk of bleeding; horizontal line at 0). The decision curves analysis showed that the cancer score use is roughly identical to the original score in patients without cancer but superior to the original score in patients with cancer.

## 4 Discussion

The current study from the BleeMACS registry that includes patients admitted with AMI with and without a diagnosis of cancer shows that the PRECISE‐DAPT cancer score that was developed by adding cancer as a binary variable has a better discrimination ability and classifies 95% of patients with cancer as HBR compared to the original PRECISE‐DAPT score, has moderate discriminative ability and classifies just 63% of patients with cancer as HBR. While the net benefits of using the original PRECISE‐DAPT score and the PRECISE‐DAPT cancer score were comparable in patients without cancer, the PRECISE‐DAPT cancer provides better precision in identifying patients with cancer and are at higher vulnerability for bleeding.

Patients presenting with AMI and a concomitant cancer diagnosis have a higher risk of bleeding than patients without cancer. For instance, data from the nationwide Swedish quality registry and the BleeMACS registry showed that cancer is a strong predictor of major bleeding in AMI patients [18, 27]. Previous studies from the United States on the outcomes of patients with cancer who undergo PCI showed that patients with cancer have an increased risk for readmissions with AMI or bleeding depending on cancer type [13, 14]. Therefore, the Academic Research Consortium for High Bleeding Risk (ARC‐HBR) considers active malignancy as a major criteria for HBR at the time of PCI [11]. Nevertheless, most bleeding risk assessment tools do not include active malignancy as a risk factor, probably because patients with cancer are excluded from the clinical trials where the scores were derived from. To address this gap, the PRECISE‐DAPT cancer score was developed by adding cancer diagnosis as a predictor for major bleeding [16]. The PRECISE‐DAPT cancer score was internally validated in a population‐based study derived from the British MINAP registry which showed that the PRECISE‐DAPT cancer score has better performance than the original PRECISE‐DAPT score, particularly in patients without cancer [16]. To the best of our knowledge, this is the first study that validates the PRECISE‐DAPT cancer score externally.

Our study shows that the PRECISE‐DAPT cancer score outperforms the original PRECISE‐DAPT score in patients with cancer, without undermining the PRECISE‐DAPT performance in patients without cancer. According to the original PRECISE‐DAPT score, only 63% of patients with cancer would have been considered HBR compared to 95% of patients if the cancer score was used. This indicates that around 32% of patients with cancer who should be considered HBR and receive a shorter duration of DAPT would receive DAPT for longer periods if the original score was used, exposing them to increased risks of major bleeding.

The Academic Research Consortium for High Bleeding Risk (ARC‐HBR) defines HBR (major bleeding rate more than 4% at the first year), hence, the current guidelines recommend classification of all patients with active malignancy as HBR patients [11]. The current study suggests that the use of the PRECISE‐DAPT cancer score may provide a more accurate personalized risk stratification rather than assuming that all patients with cancer are HBR. In fact, the PRECISE‐DAPT cancer classifies 5% of the patients with cancer as moderate bleeding risk who should qualify for a standard duration of DAPT. These findings indicate that not all patients with cancer should be classified as HBR, and the PRECISE‐DAPT cancer can be a valuable tool to identify patients with cancer with low‐moderate bleeding risk. The ability of the PRECISE‐DAPT cancer score to classify the bleeding risk in patients with and without cancer makes it an efficient, convenient tool that will help clinicians make informed decisions about the use and duration of DAPT in AMI patients with and without cancer.

This study should be interpreted in the context of several potential limitations. This study did not include patients undergoing PCI for chronic coronary syndrome nor assessed the impact of changes to the bleeding risk factors such as DAPT discontinuation or switching between antiplatelet drugs. We were not able to assess the score performance in prediction of minor bleeding because it was not captured systematically, as minor bleeding typically does not lead to hospital admission or blood transfusion. We were not able to put into consideration some comorbidities and bleeding risk factors like cancer type, cancer treatment, and stage, thrombocytopenia, and use of nonsteroidal anti‐inflammatory drugs.

To conclude, this study supports that the PRECISE‐DAPT cancer score confers a better performance and discrimination ability than the original PRECISE‐DAPT score in patients with cancer. The PRECISE‐DAPT cancer score would help cardiologists make informed decisions about the bleeding risk in patients with cancer and presenting with AMI.

## Conflicts of Interest

The authors declare no conflicts of interest.

## Supporting information

supmat.

## Data Availability

The data underlying this article cannot be shared publicly for the privacy of individuals that participated in the study. The data will be shared at reasonable requests to the corresponding author.

## References

[1] I. R. Dégano , V. Salomaa , G. Veronesi , et al., “Twenty‐Five‐Year Trends in Myocardial Infarction Attack and Mortality Rates, and Case‐Fatality, in Six European Populations,” Heart 101, no. 17 (September 2015): 1413–1421. 25855798 10.1136/heartjnl-2014-307310

[2] C. E. DeSantis , C. C. Lin , A. B. Mariotto , et al., “Cancer Treatment and Survivorship Statistics, 2014,” CA: A Cancer Journal for Clinicians 64, no. 4 (August 2014): 252–271. 24890451 10.3322/caac.21235

[3] M. A. Velders , E. Hagström , and S. K. James , “Temporal Trends in the Prevalence of Cancer and Its Impact on Outcome in Patients With First Myocardial Infarction: A Nationwide Study,” Journal of the American Heart Association 9, no. 4 (February 2020): e014383. 32067596 10.1161/JAHA.119.014383 PMC7070202

[4] N. V. Pothineni , N. N. Shah , Y. Rochlani , et al., “Temporal Trends and Outcomes of Acute Myocardial Infarction in Patients With Cancer,” Annals of Translational Medicine 5, no. 24 (December 2017): 482. 29299444 10.21037/atm.2017.11.29 PMC5750289

[5] Z. Raisi‐Estabragh , O. Kobo , P. Freeman , et al., “Temporal Trends in Disease‐Specific Causes of Cardiovascular Mortality Amongst Patients With Cancer in the USA Between 1999 and 2019,” European Heart Journal‐Quality of Care and Clinical Outcomes 9, no. 1 (December 2022): 54–63. 35435219 10.1093/ehjqcco/qcac016 PMC9745666

[6] O. Kobo , Z. Raisi‐Estabragh , S. Gevaert , et al., “Impact of Cancer Diagnosis on Distribution and Trends of Cardiovascular Hospitalizations in the USA Between 2004 to 2017,” European Heart Journal‐Quality of Care and Clinical Outcomes 8, no. 7 (November 2022): 787–797. 35913736 10.1093/ehjqcco/qcac045 PMC9603542

[7] Z. Raisi‐Estabragh , O. Kobo , T. López‐Fernández , et al., “Social Disparities in Cardiovascular Mortality of Patients With Cancer in the USA Between 1999 and 2019,” International Journal of Cardiology: Cardiovascular Risk and Prevention 19 (December 2023): 200218. 37841449 10.1016/j.ijcrp.2023.200218 PMC10568337

[8] J. P. Collet , H. Thiele , E. Barbato , et al., “2020 ESC Guidelines for the Management of Acute Coronary Syndromes in Patients Presenting Without Persistent ST‐Segment Elevation,” European Heart Journal 42, no. 14 (April 2021): 1289–1367. 32860058 10.1093/eurheartj/ehaa575

[9] G. N. Levine , E. R. Bates , J. A. Bittl , et al., “2016 ACC/AHA Guideline Focused Update on Duration of Dual Antiplatelet Therapy in Patients With Coronary Artery Disease: A Report of the American College of Cardiology/American Heart Association Task Force on Clinical Practice Guidelines,” Journal of Thoracic and Cardiovascular Surgery 152, no. 5 (November 2016): 1243–1275. 27751237 10.1016/j.jtcvs.2016.07.044

[10] C. Montalto , F. Costa , S. Leonardi , et al., “Dual Antiplatelet Therapy Duration After Percutaneous Coronary Intervention in Patients With Indication to Oral Anticoagulant Therapy. A Systematic Review and Meta‐Analysis of Randomized Controlled Trials,” European Heart Journal–Cardiovascular Pharmacotherapy 9, no. 3 (April 2023): 220–230. 36427063 10.1093/ehjcvp/pvac065

[11] P. Urban , R. Mehran , R. Colleran , et al., “Defining High Bleeding Risk in Patients Undergoing Percutaneous Coronary Intervention: A Consensus Document From the Academic Research Consortium for High Bleeding Risk,” European Heart Journal 40, no. 31 (August 2019): 2632–2653. 31116395 10.1093/eurheartj/ehz372 PMC6736433

[12] T. López‐Fernández , A. R. Lyon , and J. Herrmann , “2022 ESC Guidelines on Cardio‐Oncology: How Can We Improve the Cardiovascular Health of Patients With Cancer and Cancer Survivors?,” European Heart Journal & Cardiovascular Pharmacotherapy 9, no. 1 (January 2023): 4–5. 10.1093/ehjcvp/pvac051 PMC9923207 36107817

[13] C. S. Kwok , C. W. Wong , E. Kontopantelis , et al., “Percutaneous Coronary Intervention in Patients With Cancer and Readmissions Within 90 Days for Acute Myocardial Infarction and Bleeding in the USA,” European Heart Journal 42, no. 10 (March 2021): 1019–1034. 33681960 10.1093/eurheartj/ehaa1032

[14] J. E. Potts , C. A. Iliescu , J. C. Lopez Mattei , et al., “Percutaneous Coronary Intervention in Cancer Patients: A Report of the Prevalence and Outcomes in the United States,” European Heart Journal 40, no. 22 (June 2019): 1790–1800. 30500952 10.1093/eurheartj/ehy769

[15] F. Costa , D. van Klaveren , S. James , et al., “Derivation and Validation of the Predicting Bleeding Complications in Patients Undergoing Stent Implantation and Subsequent Dual Antiplatelet Therapy (PRECISE‐DAPT) Score: A Pooled Analysis of Individual‐Patient Datasets From Clinical Trials,” Lancet 389, no. 10073 (March 2017): 1025–1034. 28290994 10.1016/S0140-6736(17)30397-5

[16] M. Dafaalla , F. Costa , E. Kontopantelis , et al., “Bleeding Risk Prediction After Acute Myocardial Infarction‐Integrating Cancer Data: The Updated PRECISE‐DAPT Cancer Score,” European Heart Journal 45, no. 34 (September 2024): 3138–3148. 39016180 10.1093/eurheartj/ehae463 PMC11379492

[17] S. Raposeiras‐Roubín , J. Faxén , A. Íñiguez‐Romo , et al., “Development and External Validation of a Post‐Discharge Bleeding Risk Score in Patients With Acute Coronary Syndrome: The BleeMACS Score,” International Journal of Cardiology 254 (March 2018): 10–15. 29407077 10.1016/j.ijcard.2017.10.103

[18] M. Iannaccone , F. D'Ascenzo , P. Vadalà , et al., “Prevalence and Outcome of Patients With Cancer and Acute Coronary Syndrome Undergoing Percutaneous Coronary Intervention: A BleeMACS Substudy,” European Heart Journal. Acute Cardiovascular Care 7, no. 7 (October 2018): 631–638. 28593789 10.1177/2048872617706501

[19] F. Costa , D. Van Klaveren , A. Colombo , et al., “A 4‐Item PRECISE‐DAPT Score for Dual Antiplatelet Therapy Duration Decision‐Making,” American Heart Journal 223 (May 2020): 44–47. 32151822 10.1016/j.ahj.2020.01.014

[20] A. Wester , M. A. Mohammad , G. Olivecrona , J. Holmqvist , T. Yndigegn , and S. Koul , “Validation of the 4‐Item PRECISE‐DAPT Score: A SWEDEHEART Study,” Journal of the American Heart Association 10, no. 20 (October 2021): e020974. 34612051 10.1161/JAHA.121.020974 PMC8751860

[21] J. S. Birkhead , C. F. M. Weston , and R. Chen , “Determinants and Outcomes of Coronary Angiography After Non‐ST‐Segment Elevation Myocardial Infarction. A Cohort Study of the Myocardial Ischaemia National Audit Project (MINAP),” Heart 95, no. 19 (October 2009): 1593–1599. 19508971 10.1136/hrt.2008.164426

[22] M. Rashid , N. Curzen , T. Kinnaird , et al., “Baseline Risk, Timing of Invasive Strategy and Guideline Compliance in NSTEMI: Nationwide Analysis From MINAP,” International Journal of Cardiology 301 (February 2020): 7–13. 31810815 10.1016/j.ijcard.2019.11.146

[23] M. Rashid , E. Kontopantelis , T. Kinnaird , et al., “Association Between Hospital Cardiac Catheter Laboratory Status, Use of an Invasive Strategy, and Outcomes After NSTEMI,” Canadian Journal of Cardiology 36, no. 6 (June 2020): 868–877. 32146069 10.1016/j.cjca.2019.10.010

[24] M. Dafaalla , H. Abdel‐Qadir , C. P. Gale , et al., “Outcomes of ST Elevation Myocardial Infarction in Patients With Cancer: A Nationwide Study,” European Heart Journal‐Quality of Care & Clinical Outcomes 9, no. 8 (December 2023): 806–817. 36921979 10.1093/ehjqcco/qcad012

[25] E. Herrett , L. Smeeth , L. Walker , and C. Weston , “Group on Behalf of the MA. The Myocardial Ischaemia National Audit Project (MINAP),” Heart 96, no. 16 (August 2010): 1264–1267. 20659944 10.1136/hrt.2009.192328 PMC3505836

[26] A. J. Vickers , B. van Calster , and E. W. Steyerberg , “A Simple, Step‐by‐Step Guide to Interpreting Decision Curve Analysis,” Diagnostic and Prognostic Research 3 (2019): 18. 31592444 10.1186/s41512-019-0064-7 PMC6777022

[27] M. A. Velders , E. Hagström , and S. K. James , “Temporal Trends in the Prevalence of Cancer and Its Impact on Outcome in Patients With First Myocardial Infarction: A Nationwide Study,” Journal of the American Heart Association 9, no. 4 (February 2020): e014383. 32067596 10.1161/JAHA.119.014383 PMC7070202

