# Supplementary material for: External Validation of the PRECISE‐DAPT Cancer Score in Patients With Acute Myocardial Infarction
Source: Catheter Cardiovasc Interv. 2025 Jul 22;106(3):1912–9. doi: 10.1002/ccd.70040 (PMC12412353; doi:10.1002/ccd.70040)
Supplement: Supplementary file 1 — supmat. [file CCD-106-1912-s001.docx]

**Supplements**

Supplementary figure 1: HR of bleeding of the PRECISE DAPT original score vs the PRECISE DAPT cancer score


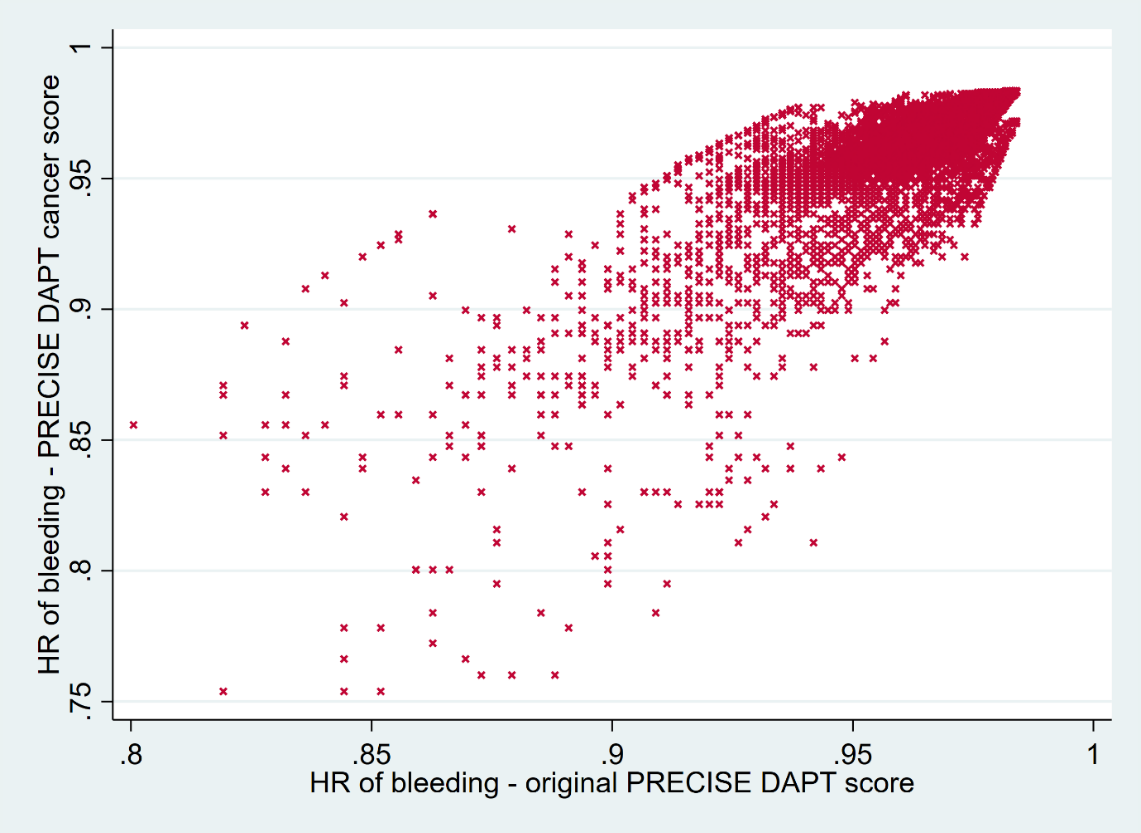


Supplementary figure 2: Cumulative incidence of bleeding events according to the PRECISE-DAPT cancer score
